# Promoter variants of *Xa23* alleles affect bacterial blight resistance and evolutionary pattern

**DOI:** 10.1371/journal.pone.0185925

**Published:** 2017-10-05

**Authors:** Hua Cui, Chunlian Wang, Tengfei Qin, Feifei Xu, Yongchao Tang, Ying Gao, Kaijun Zhao

**Affiliations:** National Key Facility for Crop Gene Resources and Genetic Improvement (NFCRI), Institute of Crop Science, Chinese Academy of Agriculture Sciences (CAAS), Beijing, China; Fujian Agriculture and Forestry University, CHINA

## Abstract

Bacterial blight, caused by *Xanthomonas oryzae* pv. *oryzae* (*Xoo*), is the most important bacterial disease in rice (*Oryza sativa* L.). Our previous studies have revealed that the bacterial blight resistance gene *Xa23* from wild rice *O*. *rufipogon* Griff. confers the broadest-spectrum resistance against all the naturally occurring *Xoo* races. As a novel executor *R* gene, *Xa23* is transcriptionally activated by the bacterial avirulence (Avr) protein AvrXa23 via binding to a 28-bp DNA element (*EBE*_AvrXa23_) in the promoter region. So far, the evolutionary mechanism of *Xa23* remains to be illustrated. Here, a rice germplasm collection of 97 accessions, including 29 rice cultivars (*indica* and *japonica*) and 68 wild relatives, was used to analyze the evolution, phylogeographic relationship and association of *Xa23* alleles with bacterial blight resistance. All the ~ 473 bp DNA fragments consisting of promoter and coding regions of *Xa23* alleles in the germplasm accessions were PCR-amplified and sequenced, and nine single nucleotide polymorphisms (SNPs) were detected in the promoter regions (~131 bp sequence upstream from the start codon ATG) of *Xa23/xa23* alleles while only two SNPs were found in the coding regions. The SNPs in the promoter regions formed 5 haplotypes (*Pro-A*, *B*, *C*, *D*, *E*) which showed no significant difference in geographic distribution among these 97 rice accessions. However, haplotype association analysis indicated that *Pro-A* is the most favored haplotype for bacterial blight resistance. Moreover, SNP changes among the 5 haplotypes mostly located in the *EBE/ebe* regions (*EBE*_AvrXa23_ and corresponding *ebes* located in promoters of *xa23* alleles), confirming that the *EBE* region is the key factor to confer bacterial blight resistance by altering gene expression. Polymorphism analysis and neutral test implied that *Xa23* had undergone a bottleneck effect, and selection process of *Xa23* was not detected in cultivated rice. In addition, the *Xa23* coding region was found highly conserved in the Oryza genus but absent in other plant species by searching the plant database, suggesting that *Xa23* originated along with the diversification of the Oryza genus from the grass family during evolution. This research offers a potential for flexible use of novel *Xa23* alleles in rice breeding programs and provide a model for evolution analysis of other executor *R* genes.

## Introduction

Plants could co-evolve in response to changes of pathogens [1]. Resistance of a plant to a pathogen with Avr protein effectors, which can be secreted and internalized into the plant cells through type-III secretion pathway, is due to the recognition by plant surveillance system [2]. Accordingly, plants have evolved resistance (*R*) genes to interact with their cognate *avr* genes and activate host immune responses [3, 4]. However, intensive diversifying selection allowed the pathogen to diversify its effector genes and escape recognition by the plant resistance gene, resulting in loss of the *R* gene-mediated resistance [1]. The constant interactions between hosts and pathogens are thought to play an important role in the evolution of *R* genes in plants and *avr* genes in pathogens. Thus, an in-depth study of the molecular evolution of *R* genes will be of significance for identifying novel or “hidden” resistant alleles and unravelling the role of pathogen-imposed selection of *R* genes [5–8].

The polymorphism and molecular evolution of plant *R* genes have been extensively studied [9–13] and found three kinds of distinctly regular patterns [8, 14–16]. The first is conserved type with little variation in the population or species, which identifies conservative *avr* genes and accounts for 63% of the *R* genes of rice genome, such as *Pi-ta* [17]. The second is the opposite type with abundant variation in population or species, which shares the mutations among different alleles by recombination and identifies conservative and non-conservative *avr* genes, such as *Rpp13* and *Rpp8* [18, 19]. The third is present and absent *R* genes, such as *Rpm1* and *Rpm5* [20]. Although, in contrast to plants, pathogens have always been the dominant force in such arm-races, it may be a feasible way to identify the disease resistance genes against the pathogen by following the variation pattern. However, previous studies have mostly focused on nucleotide-binding site leucine-rich repeat (*NBS-LRR*) type *R* genes and there is no in-depth investigation on genetic diversity analysis of the plant executor *R* genes which is triggered by activator-like effector (TALE) to activate defense response in plants [21–25].

Bacterial blight (BB) caused by *Xanthomonas oryzae* pv. *oryzae* (*Xoo*) is one of the most harmful and overwhelming diseases in rice [25]. This disease is rampant in humid tropic and temperate regions and reduces rice production up to 50%-90% [26]. To date, three executor *R* genes have been identified and cloned in rice [25]. *Xa10* and *Xa27* are two executor *R* genes, encoding 126- and 113-aa proteins respectively, with no conserved domains of any known *R* gene product [21, 24]. *Xa27* is the first cloned executor *R* gene in plants that provides disease resistance to *Xoo* strains obtained from different Asian countries [21, 27] and the XA27 protein depends on its N-terminal signal-anchor-like sequence to localize to the apoplast [28]. The resistant allele *Xa27* in the rice line IRBB27 and the susceptible allele *xa27* in IR24 contain the identical coding sequences but only the resistant allele expresses *Xa27* upon infection by *Xoo* strains expressing AvrXa27, a natural TALE protein [21]. Expressing of *Xa27* depends on the interaction between its promoter and AvrXa27. Similarly, *Xa10* gene in rice confers race-specific but narrow-spectrum resistance to *Xoo* strains containing transcription activator-like effector (TALE) gene *avrXa10* [29, 30]. Unlike *Xa27*, the tested susceptible cultivars (Nipponbare, IR24, IRBB5 and 93–11) do not have an identical open reading frame (ORF) of *Xa10* [24].

The *Xa23*, a recently cloned BB resistance executor *R* gene identified from wild rice (*O*. *rufipogon*) is transcriptionally activated by AvrXa23, a transcription activator-like effector (TALE) encoded in *Xoo* strains [31]. Because the *avrXa23* is widely present in all tested naturally occurring *Xoo* strains, the rice with *Xa23* exhibit extremely broad-spectrum resistance against bacterial blight [32]. *Xa23* is an intronless gene encoding a 113-amino acid protein with three predicted trans-membrane domains [25]. CBB23, a near isogenic line obtained through transfer of *Xa23* from the wild rice into cultivated *indica* rice (O. *sativa* ssp. *indica*) variety JG30, confers the broadest resistance to *Xoo* strains [32]. Comparatively, the recessive allele *xa23* in JG30 which is susceptible to *Xoo* strains contains the identical coding region of *Xa23*. The main nucleotide polymorphism between these two alleles (*Xa23* in CBB23 and *xa23* in JG30) is located in their respective promoter regions. The 28-bp *EBE*_AvrXa23_ (AvrXa23 binding element) is present in the promoter of *Xa23* allele and can interact with AvrXa23. Through the analysis of polymorphisms in coding and promoter regions of *Xa23/xa23* alleles in cultivated rice and their wild relatives, we would investigate whether the promoter mutation of *Xa23* plays an important role in resistant varieties and study the origin of this allele and trace its ancestry among the genetically divergent subpopulations of rice.

In this work, we analyzed nucleotide diversity in the promoter regions (-131 bp upstream sequence from the start codon ATG) and coding regions of *Xa23/xa23* alleles from 97 different rice varieties/accessions including 29 cultivated rice (consist of 20 *indica* and 9 *japonica*) and 68 wild relatives (consist of 41 *O*. *rufipogon*, 10 *O*. *nivara*, 14 *O*. *officinalis wall*, 1 *O*. *latifolia desy*, 1 *O*. *glumaepatula* and 1 *O*. *alta swallen*). The major objectives of this study were: (1) to find out homologs of *Xa23* across different plant species; (2) to analyze the nucleotide diversity of *Xa23/xa23* alleles; (3) to detect the association between *Xa23/xa23* haplotypes and BB resistance as well as the haplotypes distribution in rice; and (4) take the evolutionary model of *Xa23* as a direction or route map for other plant executor *R* genes to develop more BB resistant resources and produce valuable materials for rice breeding.

## Materials and methods

### Plant materials and growth conditions

A total of 97 rice varieties/accessions (20 *indica*, 9 *japonica* and 68 wild relatives) (S1 Table) were used for sequencing and nucleotide diversity analysis of *Xa23/xa23* alleles. The 97 rice materials are in forms of DNA, rice leaves and rice seed samples. Among them, seeds of 51 rice materials were planted in a paddy field in Beijing (39°54’N, 116°23’E), China.

### Database search and identification of *Xa23* homologs

We blasted the *EBE*_AvrXa23_ (28-bp) and *ORF* (342-bp) sequences of *Xa23* allele from CBB23 in NCBI (National Center for Biotechnology Information, http://www.Ncbi.nlm.nlh.gov), Gramene (A comparative resource for plants, http://www.gramene.org/) and RGAP (Rice Genome Annotation Project, http://rice.plantbiology.msu.edu/index.shtml). We also searched the *EBE*_AvrXa23_ sequence in Rice SNP-Seek Database (http://www.oryzasnp.org/iric-portal) which consist of Oracle database having a total number of rows with SNP genotypes near to 60 billion (20 M SNPs × 3 K rice lines) and web interface for convenient querying [33, 34] and Phytozome (http://phytozome.jgidoe.gov/pz/portal.html). Altogether 39 plant species were selected to retrieve the *EBE*_AvrXa23_ and *Xa23* coding sequences. The *Xa23* in CBB23 and *xa23* in JG30 were used as reference sequences [25].

### DNA extraction, PCR and sequencing

**Fig 1:**
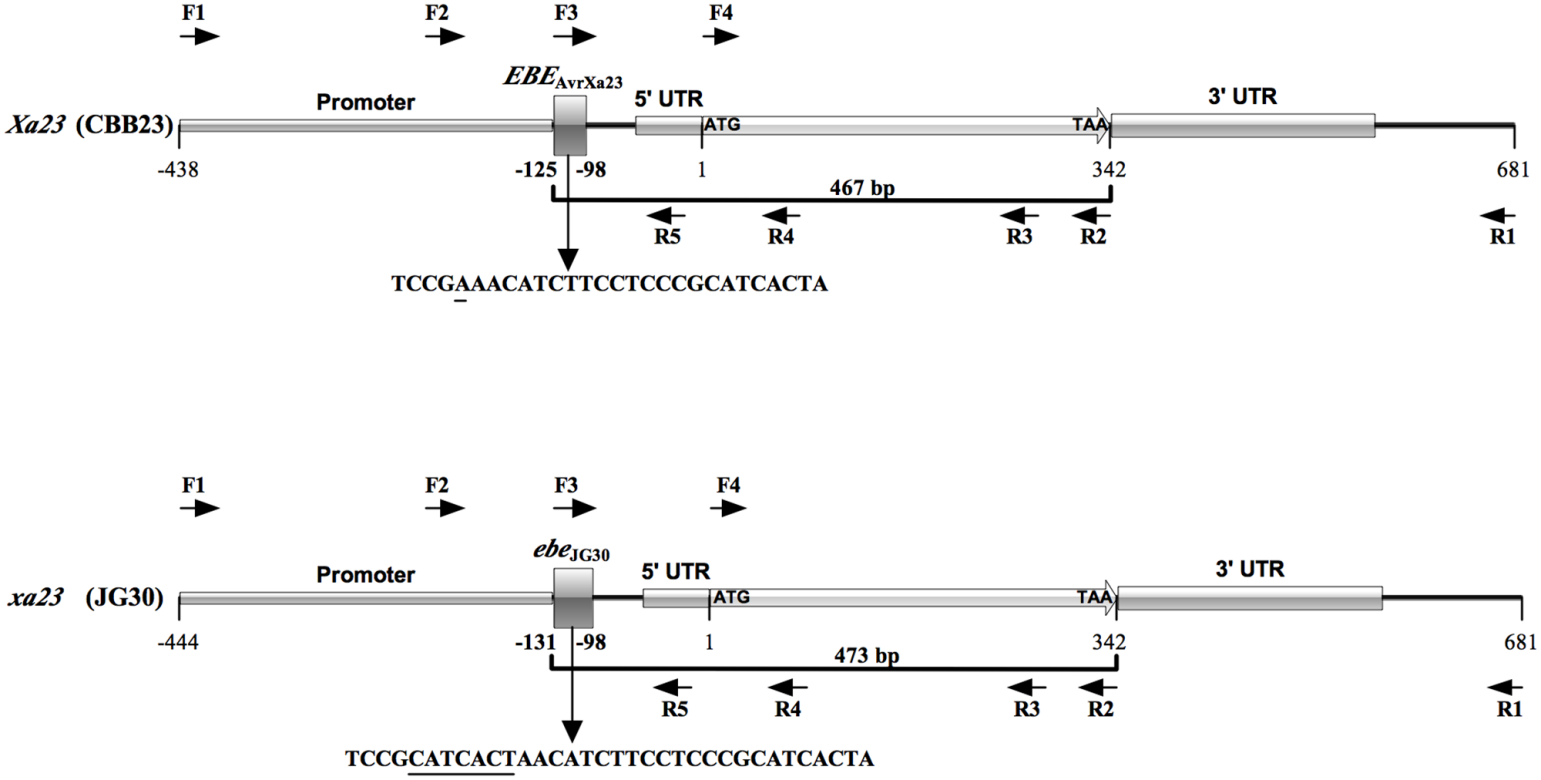
A schematic presentation of gene structure of *Xa23/xa23* alleles and primer positions. The resistant *Xa23* allele in CBB23 and susceptible *xa23* allele in JG30 were used to show the gene structure and primer locations. Overlapping primers were designed to amplify DNA fragments covering the *EBE*_AvrXa23_ in CBB23, the corresponding *ebe* region in JG30 and their coding regions; arrows represent the locations and the orientations of the primers. The numbers indicate the positions and lengths of different regions in the two alleles. The alignment regions are the sequences ranging from the first nucleotide of the *EBE/ebe* to the last nucleotide of the stop codon. Because of the *ebe*_JG30_ has 7-bp polymorphic nucleotides (6-bp insertion and 1-bp substitution, underlined) compared to the *EBE*_AvrXa23_, the corresponding alignment regions in length are 467-bp in *Xa23* of CBB23 and 473-bp in *xa23* of JG30, respectively.

Genomic DNA was extracted from ~ 100 mg of rice leaves by using CTAB method [35]. Based on the known sequence of *Xa23* in CBB23, considering that the flanking sequences of *Xa23* gene are highly complex (one transposon and four repeat sequences) may affect the amplification results, we used the Primer Premier 5.0 (Premier Biosoft, Palo Alto, CA) to design several pairs of primers (Fig 1, S1 Fig, S2 Table) to make the amplification results more accurate. Depending on the positions of the forward and reverse primers, they can be matched with each other for amplification of the corresponding *Xa23* alleles in different *Oryza* species. Polymerase Chain Reaction (PCR) was performed in a Veriti 96-Well Thermal Cycler using highly-efficient KOD polymerase in a total volume of 20 μl reaction mixture. Briefly, reaction mixture contained 100 ng of genomic DNA, 0.5 μM of each primer, 0.2 mM of each dNTP, 2× PCR buffer (10 mM Tric-Hcl, pH 8.8, 1.5 mM MgCl_2_) 10 μL, and 0.2 unit (1U/μL) of KOD. The PCR profile consists of 3 min initial denaturation at 94°C, 35 cycles of amplification with 30 s DNA denaturation at 94°C, 30 s annealing at 60°C and a final elongation at 72°C with 30 s-60 s depending on the length of different fragment. Subsequently, all amplified products were visualized on 1% agarose gels and sequenced.

### Statistical analysis

The genomic sequences of *Xa23* alleles obtained from 97 rice accessions were aligned and assembled by *Clustal X* version 2.0 [36] and *BioEdit* [37, 38]. The aligned file was used as an input format for analysis into *DnaSP* version 5.0 [39]. The number of polymorphic sites, including single nucleotide polymorphisms (SNPs), insertions and deletions (InDels) in promoter and coding regions were determined according to *DnaSP* version 5.0 [39]. Nucleotide diversity was also analyzed by estimating average number of nucleotide diversity per pair (*π*) [40] and number of segregating sites (*θ*_*w*_) [41]. Different neutral test such as Tajima’s *D* [42] and Fu and Li’s *D* [43] were calculated separately for *indica*, *japonica* and wild rice by using *DnaSP* to determine whether the locus is departed from neutrality. Phylogenetic network analysis of the entire *Xa23/xa23* alleles was performed through *Network* version 4.6 [44].

### Assessment of rice resistance to *Xoo* strain PXO99^A^

The *Xoo* strain PXO99^A^ was cultured in PSA medium at 28°C for 48 hours. Bacterial suspensions (OD_600_ = 1.0) were used for inoculation on fully expanded rice leaves at the seedling stage. The pathogenicity assay on the PXO99^A^ was performed using the leaf-clipping method [45]. Disease symptoms were recorded two weeks after inoculation and measured by lesion length [46].

## Results

### Homologs search of *Xa23* allele in different species

Our previous work indicated that *Xa23* is a single copy gene in the rice variety CBB23 and the 28-bp *EBE*_AvrXa23_ localized in the *Xa23* promoter is the core element for interaction with the pathogen effector AvrXa23. To find out the homologs of *Xa23* gene across different species, we performed a blast with *CDS*_*Xa23*_ (342-bp) and *EBE*_AvrXa23_ (28-bp) sequences separately in Gramene (http://www.gramene.org/), NCBI (National Center for Biotechnology Information, https://blast.ncbi.nlm.nih.gov/Blast.cgi) and RGAP (Rice Genome Annotation Project, http://rice.plantbiology.msu.edu/index.shtml). As a result, we found *Xa23* homologous only in the *Oryza* genus; no significant homologous sequences with an *E-value* < 1 has been found in other plant species. In *Oryza*, the coding regions of *Xa23* alleles ranged from 327 bp to 492 bp in length and the nucleotide sequence identity compared with *Xa23* in CBB23 ranged from 88% to 100% (Table 1, S2 Fig), indicating a highly conserved coding region of *Xa23* alleles. Moreover, we didn’t find any *EBE*_AvrXa23_ sequence present in cultivated rice by Rice SNP-Seek Database retrieval, suggesting that the *EBE*_AvrXa23_ is absent in the cultivated rice surveyed.

**Table 1:** Summary of sequence comparison among the coding regions of *Xa23* alleles in *Oryza* species with reference to CBB23.

Species & Accession	Coding region (bp)		Protein (a.a.)	
	Total	Identity (%)	Total	Identity (%)
*Oryza sativa indica ASM465v1*	342	99	113	100
*Oryza sativa japonica IRGSP-1*.*0*	342	100	113	100
*Oryza rufipogon OR_W1943*	333	100	110	100
*Oryza glumaepatula ALNU02000000*	342	99	113	99
*Oryza meridionalis_v1*.*3*	342	99	113	99
*Oryza longistaminata_v1*.*0*	339	88	112	77
*ABB94457*	492	100	163	100
*Oryza barthii_v1*	336	88	110	80
*Oryza glaberrima AGI1*.*1*	330	88	109	81
*Oryza punctata AVCL00000000*	327	88	108	83

### Polymorphism and haplotypes of *Xa23* alleles

**Fig 2:**
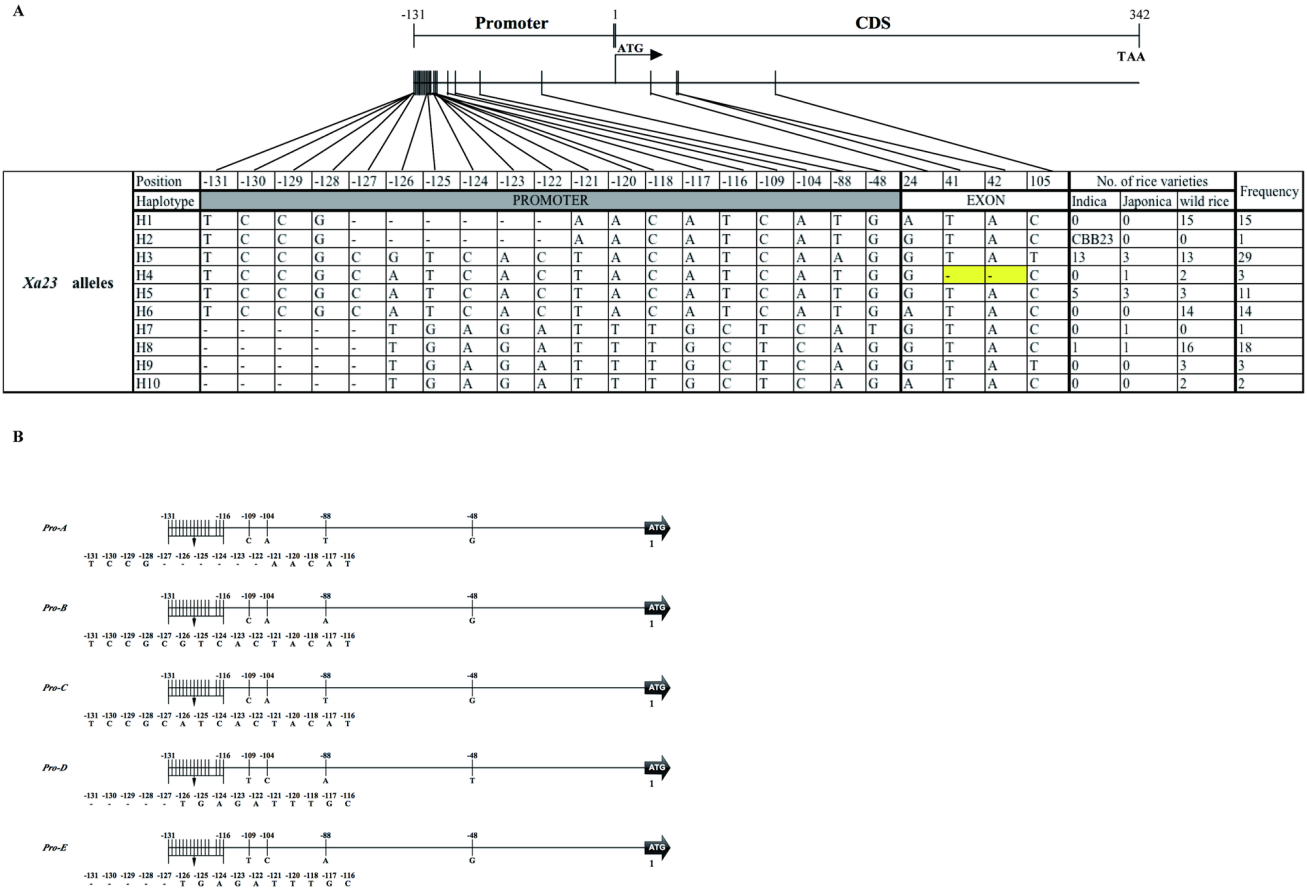
Haplotype analysis of the *Xa23* gene region in the 97 rice varieties/accessions. **(A)** The *Xa23/xa23* alleles contain the sequence covering promoter region (-131 bp upstream sequences from ATG start codon) and the coding region. The entire aligned length of the 473-bp genome sequence is shown in graphic on the top. The numbers on the top row shows the positions (cf. ATG start codon) of nucleotide polymorphisms in the region starting from the *EBE/ebe* to the stop codon TAA. The “-” indicate deletions, yellow represents missense mutations in the coding region. Ten haplotypes (H1-H10) were detected in the 97 representative rice varieties/accessions, which consist of 22 *indica*, 7 *japonica* and 68 wild rice. The total number of every haplotype and the number of every haplotype in different rice species are shown in the right columns. (B) Haplotypes in the promoter regions of *Xa23* alleles. Five haplotypes were formed by 9 SNPs and 2 InDels in the *Xa23/xa23* promoter regions.

We collected 97 representative rice varieties/accessions (S1 Table) to amplify the *Xa23* alleles by using overlapping gene-specific primers (Fig 1, S2 Table). The *Xa23/xa23* alleles from the 97 rice accessions were sequenced and aligned for their nucleotide diversity analysis. Including sites with alignment gaps, the length of total alignment (sequence starting from the *EBE/ebe* to the stop codon TAA, Fig 1) is 473 bp. Comparative analysis in *DnaSP*, excluding sites with gaps/missing data (in total 461 available sites), 11 SNPs and 3 insertions and deletions (InDels) events were detected in the aligned 473 bp (Fig 2A). Varied DNA polymorphisms were observed in promoter and coding regions of the *Xa23* alleles (Table 2).

**Table 2:** Polymorphism and neutral test in different regions of *Xa23/xa23* alleles.

Species	*Xa23* alleles	Total sites (excluding sites with gaps)	Number of haplotype	S	InDel	π	θ_W_	Fu and Li's *D*	Tajima' *D*	*K*	Ka/Ks(Jukes & Cantor)
**Whole population**	entire gene	461	9	11	3	0.00855	0.00464	0.76414	2.21473*	3.944	0.00864
	coding region	340	3	2	1	0.00261	0.00114	0.68782	1.98002	0.886	0.00261
	promoter region	121	5	9	2	0.02527	0.01445	0.57301	1.87651	3.058	0.02616
**Indica**	entire gene	463	4	9	0	0.0035	0.00548	-2.23543	-1.24143	1.621	0.00352
	coding region	342	2	1	0	0.0014	0.00082	0.64952	1.26176	0.47895	0.0014
	promoter region	121	4	8	0	0.00944	0.01864	-2.61649*	-1.66331	1.14211	0.00966
**Japonica**	entire gene	466	4	14	2	0.01264	0.01184	1.28707	0.32335	5.88889	0.01284
	coding region	340	2	1	1	0.00147	0.00108	0.8404	0.98627	0.5	0.00147
	promoter region	126	4	13	1	0.04277	0.04088	1.26193	0.22156	5.38889	0.04544
**Wild rice**	entire gene	461	7	10	3	0.00938	0.00453	1.38919	2.90562**	4.32485	0.00948
	coding region	340	3	2	1	0.00256	0.00123	0.71782	1.79613	0.86874	0.00256
	promoter region	121	4	8	2	0.02856	0.0138	1.28344	2.75975**	3.4561	0.02964

π, nucleotide diversity (average of nucleotide differences per site between two sequences); θ, Watterson estimator (no. of segregating sites); K, average nucleotide difference; Tajima's D, Fu and Li's D, test for neutral selection.

*Significant at P<0.05;

**Significant at P<0.01.

entire region: start from EBE/ebe to the TAA stop codon (-131 bp upstream sequences from ATG start codon to the TAA stop codon); promoter region: start from EBE/ebe to the ATG start codon (-131 bp upstream sequences from ATG start codon).

Based on the detected 11 SNPs and 3 InDels events, the 97 rice varieties/accessions were divided into 10 haplotypes (H1-H10 in Fig 2, S1 Table). CBB23 was defined as haplotype H2. The wild rice accessions showed more various haplotypes, in which haplotypes H1, H6 and H9-H10 containing completely different SNPs were all represented by 68 wild relatives, while *indica* haplogroup contained only three haplotypes (H3, H5 and H8) which were defined by two SNPs at positions -126 bp and -88 bp upstream from ATG and one InDel, and haplotype H7 was only identified in *japonica* varieties. Notably, four haplotypes (H3, 4, 5 and 8) were shared in both wild and cultivated rice analyzed in this study and their distribution ratio was not significantly different (Fig 2A).

**Fig 3:**
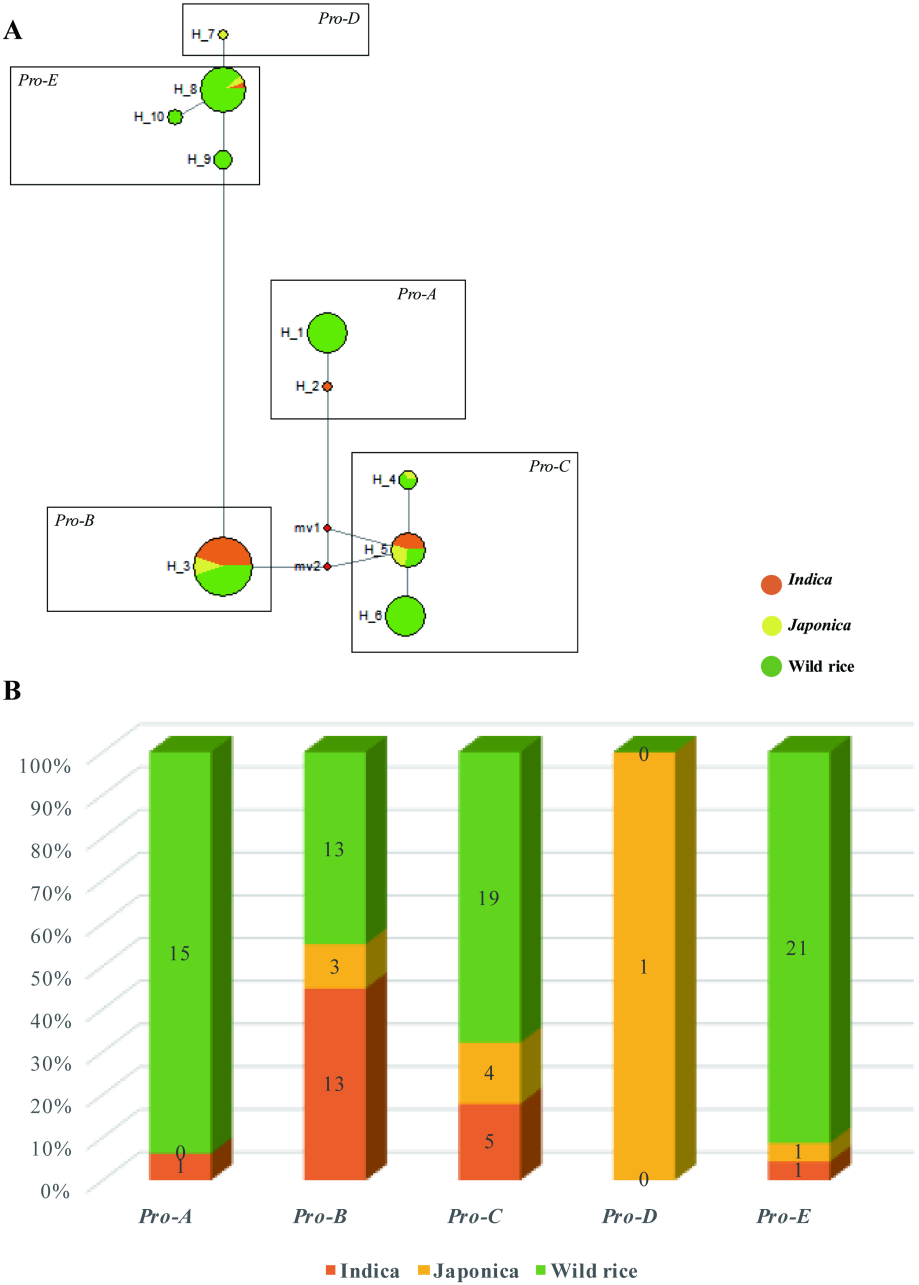
Haplotypes distribution of *Xa23* alleles. (A) Haplotype network of the *Xa23/xa23* alleles in 97 rice accessions. Haplotype frequencies are proportional to the area of the circles. The proportion of wild rice and two cultivated subgroups (*indica* and *japonica*) in each haplotype is represented by different colors. The five haplotypes (*Pro-A*, *B*, *C*, *D* and *E*) were formed based on their corresponding promoter regions. (B) The specific numbers of the five haplotypes (*Pro-A*, *B*, *C*, *D* and *E*) in 97 rice varieties/accessions.

Compared with the coding region, more variations occurred in the promoter regions (Fig 2). Five haplotypes were formed by 9 SNPs and 2 InDels events within the promoter regions of *Xa23* alleles; these haplotypes were designated *Pro-A*, *Pro-B*, *Pro-C*, *Pro-D*, *Pro-E* (Fig 2B). Furthermore, haplotypes H1 consist of 15 (15.46%) wild rice and H2 (CBB23) belonged to *Pro-A*, haplotype H3 consist of 13 (13.40%) *indica*, 3 (3.10%) *japonica* and 13 (13.40%) wild rice belonged to *Pro-B*, and H4-H6 together account 5 (5.15%) *indica*, 4 (4.12%) *japonica* and 19 (19.59%) wild rice belonged to *Pro-C*, H7 containing only 1 (1.03%) *japonica* belonged to *Pro-D* and H8-H10 consist of 1 (1.03%) *indica*, 1 (1.03%) *japonica* and 21 (21.65%) wild rice belonged to *Pro-E* (Fig 3). These results suggest that, among the five haplotypes in promoter regions, the haplotype *Pro-A* presented only in CBB23 and wild rice, whereas other four haplotypes (*Pro-B*, *C*, *D*, *E*) were distributed in both cultivated and their wild relatives; moreover, haplotype *Pro-B* seems like the major haplotype at *xa23* locus in the rice accessions used in this work.

### The association between variations in coding regions of *Xa23* alleles and BB resistance phenotypes

In order to discover the association between the nucleotide diversity and BB resistant function, we first used a representative panel of 22 rice accessions (8 *indica*, 3 *japonica* and 11 wild relatives, S3 Table) to study the relationship between coding region variations and BB resistance. The coding regions of *Xa23* alleles were highly conserved across the 22 rice accessions, and two synonymous SNPs in total were identified at 24 (S1: G/A) and 105 (S2: T/C) positions in the coding regions. The two SNPs (S1 and S2) were found to be grouped into three genotypes A1 (S1G/S2C), A2 (S1G/S2T) and A3 (S1A/S2C) (S3 Table).

**Fig 4:**
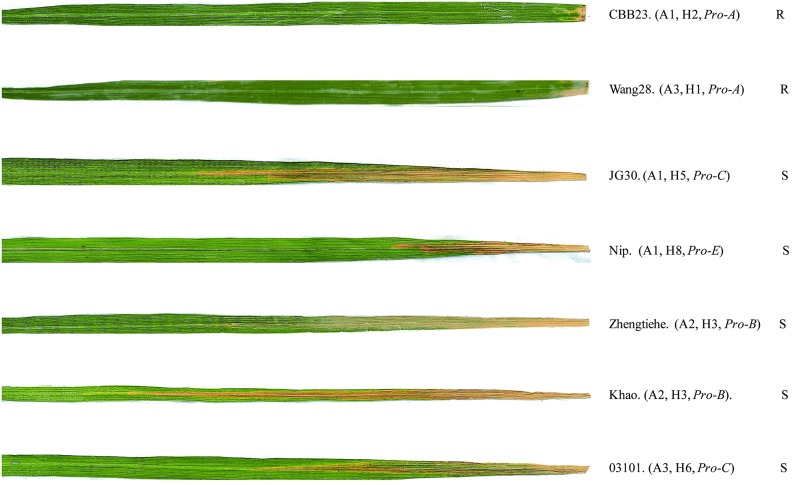
Disease reactions of representative rice accessions with different genotypes to PXO99^A^. Representative leaves of rice accessions CBB23 (H2, *Pro-A*, *indica*), Wang28 (H1, *Pro-A*, *rufipogon*), JG30 (H5, *Pro-C*, *indica*), Nipponbare (Nip. H8, *Pro-E*, *japonica*), Zhengtiehe (H3, *Pro-B*, *indica*), Khao Dawk Mali 105 (Khao. H3, *Pro-B*, *indica*) and 03101 (H6, *Pro-C*, *rufipogon*) show the resistant (R) and susceptible (S) lesions caused by *Xoo* strain PXO99^A^. Photographs were taken 14 days after inoculation. These six rice accessions contained 3 different types of coding regions, among which CBB23, JG30 and Nipponbare represent A1 (S1G/S2C), Zhengtiehe and Khao Dawk Mali 105 represent A2 (S1G/S2T), Wang28 and 03101 represent A3 (S1A/S2C).

Focused on the 22 rice accessions, we found that 3 *indica*, 2 *japonica* and 4 wild rice containing A1, 4 *indica* and 1 *japonica* containing A2 and 2 wild rice containing A3 were susceptible to *Xoo* strain PXO99^A^, whereas 1 *indica* containing A1 and 5 wild rice containing A3 all exhibited resistance (Fig 4, S3 Table). Consequently, our association study indicated that S1 and S2 have no correlation (*r*^*2*^ = 0.137, *p* = 0.13477) with the resistance/susceptibility response to PXO99^A^. Therefore, the variations in the coding regions of *Xa23* alleles did not significantly affect rice resistance to *Xoo* strain PXO99^A^.

### The association between promoter haplotypes of *Xa23* alleles and BB resistance phenotypes

Because the coding region polymorphism of *Xa23* alleles did not significantly influence the BB resistance phenotypes, we selected 51 rice varieties/accessions (13 *indica*, 5 *japonica* and 33 wild relatives, Table 3) to further study the association between the promoter region haplotypes (*Pro-A*, *B*, *C*, *D* and *E*) and BB resistance phenotypes. As a result, we found *Pro-A* is the key haplotype in conferring resistance to *Xoo* strain PXO99^A^ (Table 3, S3 Table, S3 Fig). It was also noteworthy that the major difference between susceptible haplotypes (*Pro-B*, *C*, *D* and *E*) and haplotype *Pro-A* is due to mutations in their corresponding *EBE/ebe* regions (Figs 1 and 2), which directly affect the interaction between *Xa23* and AvrXa23. Therefore, *Xa23* (containing haplotype *Pro-A*) confers the resistance to bacterial blight disease, and the different *ebe* regions contained by different *xa23* alleles may be the major reason for BB resistance elimination in rice breeding.

**Table 3:** *Xa23* haplotypes based on promoter regions and corresponding BB resistance phenotypes.

Variety/Accession	Species	Haplotype	Phenotype
CBB23	*indica*	*Pro-A*	R
93–11	*indica*	*Pro-B*	S
CO 39	*indica*	*Pro-B*	S
fr 13 a	*indica*	*Pro-B*	S
MADHABSAR	*indica*	*Pro-B*	S
Zhengtiehe	*indica*	*Pro-B*	S
Khao Dawk Mali 105	*indica*	*Pro-B*	S
da 16	*indica*	*Pro-B*	S
t 1	*indica*	*Pro-B*	S
JG30	*indica*	*Pro-C*	S
LANI KHAMA	*indica*	*Pro-C*	S
5024S	*indica*	*Pro-C*	S
Basmati 1	*indica*	*Pro-C*	S
Hongguo	*japonica*	*Pro-B*	S
66756	*japonica*	*Pro-C*	S
n 22	*japonica*	*Pro-C*	S
IRGC 5441	*japonica*	*Pro-D*	S
Nipponbare	*japonica*	*Pro-E*	S
03–8	*O*. *rufipogon*	*Pro-A*	R
03–9	*O*. *rufipogon*	*Pro-A*	R
03–19	*O*. *rufipogon*	*Pro-A*	R
04–110	*O*. *rufipogon*	*Pro-A*	R
04–102	*O*. *rufipogon*	*Pro-A*	R
03–66	*O*. *rufipogon*	*Pro-A*	R
Wang13	*O*. *rufipogon*	*Pro-A*	R
2511–1	*O*. *rufipogon*	*Pro-A*	R
Wang28	*O*. *rufipogon*	*Pro-A*	R
03–15	*O*. *rufipogon*	*Pro-A*	R
03–20	*O*. *rufipogon*	*Pro-A*	R
03–27	*O*. *rufipogon*	*Pro-A*	R
03–28	*O*. *rufipogon*	*Pro-A*	R
03–104	*O*. *rufipogon*	*Pro-A*	R
04–72	*O*. *rufipogon*	*Pro-A*	R
KHM08-37	*O*. *rufipogon*	*Pro-B*	S
KHM08-51	*O*. *rufipogon*	*Pro-B*	S
NEP09-79	*O*. *rufipogon*	*Pro-B*	S
2511–2	*O*. *rufipogon*	*Pro-C*	S
03–14	*O*. *rufipogon*	*Pro-E*	S
03–16	*O*. *rufipogon*	*Pro-E*	S
03–26	*O*. *rufipogon*	*Pro-E*	S
04–103	*O*. *rufipogon*	*Pro-E*	S
04-108S	*O*. *rufipogon*	*Pro-E*	S
HL_8	*O*. *rufipogon*	*Pro-E*	S
BC2701	*O*. *rufipogon*	*Pro-B*	S
VN04-9	*O*. *nivara*	*Pro-B*	S
VN04-10	*O*. *nivara*	*Pro-B*	S
VN07-00	*O*. *nivara*	*Pro-B*	S
VN07-10	*O*. *nivara*	*Pro-B*	S
03–101	*O*. *latifolia desy*	*Pro-C*	S
94	*O*. *alta swallen*	*Pro-C*	S
84	*O*. *glumaepatula*	*Pro-C*	S

R, resistant; S, susceptible.

### Genetic diversity of *Xa23* alleles in cultivated and wild rice

In the whole germplasm population, the average number of nucleotide difference, ‘*K*’ of the entire gene region (from the *EBE/ebe* to the stop codon TAA) was estimated to be 3.944. The genetic diversity, ‘π’ of *Xa23* alleles was 0.00855 and ‘θ_w_’ equal to 0.00464 in the promoter region (-131 bp upstream sequence from the ATG start codon) were 9- to 12- fold higher than that in coding region. The test of neutrality give a significant positive Tajima’s *D* value (*P<0*.*05*) in the entire *Xa23* genomic region. Considering the population stratification, we also tested these parameters within the three populations (*indica*, *japonica* and wild rice). The values of the π and θ_w_ in the promoter region were 6- to 22- fold, 29- to 37- fold and 11- to 11- fold higher than in coding region in *indica*, *japonica* and wild rice, separately. Moreover, compared with wild rice (average proportion of pairwise differences per base pair, π = 0.00938), a 62.69% reduction of sequence diversity was found in *O*. *sativa* ssp. *indica* varieties (π = 0.0035), while *japonica* has higher nucleotide diversity (π = 0.01264) than the *indica*. In addition, the positive Tajima’s *D* value reached a significant level in wild rice (*P*<0.05) in the promoter region and the entire gene region of wild rice. However, the Fu and Li’s *D* value showed a negative value and reached a significant level in the promoter region of *indica* varieties (-2.61649, *P*<0.05) (Table 2). These results suggest that *Xa23* was most likely undergone the bottleneck founder effect in rice during domestication and breeding.

## Discussion

Functional variations in the alleles of *R* genes which lead to different phenotypes due to polymorphisms such as SNPs and InDels of core DNA fragments. As a natural phenomenon, *R* genes usually maintain different allelic forms in the population to protect plants from evolving pathogens [47, 48]. The high-end next generation sequencing technology in combination with a wide range of genetic resources in rice, provides us with the opportunity to discover potential resistance genes or alleles and trace their evolutionary pattern as well as to reveal the key elements regulating resistance. In the present study, we have analyzed the sequence polymorphisms, phylogeographic relationship and association of *Xa23* alleles with bacterial blight resistance. This research may help to uncover genetic *Xa23* variants in cultivated and wild rice species.

Moreover, at the species level, the nucleotide diversities of *Xa23* alleles (0.0035 for *indica*, 0.01264 for *japonica* and 0.00938 for wild rice) was found to be comparatively much higher as compared to genome-wide average level of the two subspecies (π_silent_ = 0.0021 for *indica* and 0.0011 for *japonica* [49]). This may be resulted from a wider geographical distribution of the rice germplasm used in this work, as it comprised rice varieties worldwide. We also observed a much higher diversity in wild rice (*O*. *rufipogon* and *O*. *nivara*) than the cultivated *O*. *sativa*, as well as a decrease in diversity among *O*. *sativa*, which may be due to the bottleneck effect [49–51]. In addition, the non-coding regions always evolve more rapidly and show higher sequence polymorphism than coding regions under natural conditions [52], which is often discovered in most of the genes observed; and high polymorphism is expected at the locus involved in pathogen recognition [53]. In this study, among the 97 rice accessions, the nucleotide diversity in promoter of *Xa23* alleles (π = 0.02527) was about ten times than that of the coding region. The similar results appeared in *indica*, *japonica* subpopulations and wild relatives, indicating the presence of higher diversity in the promoter region of *Xa23* alleles. In details, most polymorphisms were found in the *EBE*_AvrXa23_ and the corresponding *ebe* regions which can affect the recognition between AvrXa23 encoded by *Xoo* strains and *Xa23* promoter. In addition to the *EBE*_AvrXa23_ contained only by haplotype *Pro-A* identified in the resistant *Xa23* promoter, three types of *ebe* region were found in the other four haplotypes (*Pro-B*, *C*, *D*, *E*) which accounted for a large proportion of *Xa23* haplotypes (Fig 3), and coding regions of *Xa23/xa23* alleles are highly conservative. So we speculate that the additional *ebe* regions might play a critical role when *EBE*_AvrXa23_ loses its recognition function for the *Xoo* effector AvrXa23. In this circumstance, the new Avr effector evolved in the pathogen *Xoo* may be recognized by the three types of *ebe* regions and thus stimulate the BB resistance function. Therefore, the high diversification of the promoter region provided the flexibility for *R* gene to adapt to different environments or to meet a variety of developmental requirements.

On the other hand, it will be useful to study the different alleles where amino acid changes had occurred, after fusing these newly found coding sequences with a functional *EBE*_AvrXa23_ by promoter engineering [54, 55]. Since CBB23 has been widely adopted in rice breeding programs [56, 57], and several *Xa23*-containing hybrid rice varieties have been released to famers in recent years. Thus, the method that combine the known *EBE*_AvrXa27_, *EBE*_AvrXa10_ and *EBE*_AvrXa23_ to build up a functional *EBE* element which capable of interacting with various Avr effectors is feasible and will lead to more extensive resistance and longer time application of single *R* gene.

In summary, we have analyzed the genetic polymorphism of *Xa23* in rice. This analysis is the first of its kind for executor *R* genes and may be helpful in molecular evolutionary studies and mining useful alleles for rice improvement. The main contributions and potential effects of these variants on BB resistance managements have not been analyzed. In natural population, maintenance of allelic diversity in resistance genes seems like a result of co-evolution between host and pathogen [47]. Previous researches also suggested that pathogen may be an important selective agent during the process of *R* gene evolution [58]. Therefore, following the phenotypic screening of pathogens with specific *Avr* genes, the inclusion of more individuals in the analysis will help determine the co-evolutionary relationship between pathogens and host resistance.

## Supporting information

S1 Fig Alignment of nucleotide sequences between the resistant *Xa23* allele in CBB23 and susceptible *xa23* allele in JG30. The 1720-bp sequence of *Xa23* allele in CBB23 was used as the reference. The numbers at right side indicate nucleotide positions of the CBB23 and JG30 sequences. The 28-bp *EBE*_AvrXa23_ and 34-bp *ebe*_JG30_ are highlighted in green. The 7-bp polymorphic nucleotides (6-bp insertion and 1-bp substitution) in JG30 are highlighted in purple. The coding regions of *Xa23/xa23* alleles in CBB23 and JG30 are both highlighted in blue. The primers are used for amplifying the alignment regions covering entire *EBE/ebe* and condign regions. The arrows indicate the primers and their amplification directions.
(PDF)

S2 Fig Comparison of coding regions of *Xa23/xa23* alleles in *Oryza* species. The multiple sequence alignment was constructed using CLC Sequence Viewer 7 program. The *Xa23* in CBB23 is used as a reference. The different residues are shown in red. The consensus sequence of all the *Xa23/xa23* alleles along with percentage conservation of residue is also shown. The numbers at right side indicate the length of each sequence.
(PDF)

S3 Fig Disease responses of five haplotypes of *Xa23/xa23* alleles to PXO99^A^. These 18 representative rice accessions (8 *indica*, 3 *japonica* and 7 wild relatives) were inoculated with *X*. *oryzae* pv. *oryzae* strain PXO99^A^ using leaf-clipping method and bacterial blight lesions were measured 14 days after artificial inoculation. Y-axis is showing the lesion length. The *Pro-A*, *B*, *C*, *D* and *E* at the top indicate the five haplotypes of *Xa23/xa23* alleles. W, wild rice; I, *indica*; J, *japonica*; R, resistant; S, susceptible.
(PDF)

S1 Table Summary of rice used for polymorphism and haplotype analysis. (PDF)

S2 Table Primers used for amplification and sequencing in this research. (PDF)

S3 Table The SNPs in coding regions of *Xa23* alleles and corresponding bacterial blight resistance phenotypes. (PDF)

## References

[1] Bimolata W, Kumar A, Sundaram RM, Laha GS, Qureshi IA, Reddy GA, et al Analysis of nucleotide diversity among alleles of the major bacterial blight resistance gene *Xa27* in cultivars of rice (*Oryza sativa*) and its wild relatives. Planta. 2013; 238(2): 293–305. Epub 2013/05/09. doi: 10.1007/s00425-013-1891-3 . 23652799 10.1007/s00425-013-1891-3

[2] Greenberg JT, Vinatzer BA. Identifying type III effectors of plant pathogens and analyzing their interaction with plant cells. Current opinion in microbiology. 2003; 6(1): 20–8. Epub 2003/03/05. . 12615215 10.1016/s1369-5274(02)00004-8

[3] Dangl JL, Jones JD. Plant pathogens and integrated defence responses to infection. Nature. 2001; 411(6839): 826–33. Epub 2001/07/19. doi: 10.1038/35081161 . 11459065 10.1038/35081161

[4] Chen Q, Han Z, Jiang H, Tian D, Yang S. Strong positive selection drives rapid diversification of *R*-genes in *Arabidopsis* relatives. J Mol Evol. 2010; 70(2): 137–48. Epub 2010/01/02. doi: 10.1007/s00239-009-9316-4 . 20044783 10.1007/s00239-009-9316-4

[5] McDowell JM, Woffenden BJ. Plant disease resistance genes: recent insights and potential applications. Trends Biotechnol. 2003; 21(4): 178–83. Epub 2003/04/08. doi: 10.1016/S0167-7799(03)00053-2 . 12679066 10.1016/S0167-7799(03)00053-2

[6] Tiffin P, Moeller DA. Molecular evolution of plant immune system genes. Trends in genetics: TIG. 2006; 22(12): 662–70. Epub 2006/10/03. doi: 10.1016/j.tig.2006.09.011 . 17011664 10.1016/j.tig.2006.09.011

[7] Jia Y, Bryan GT, Farrall L, Valent B. Natural variation at the *Pi-ta* rice blast resistance locus. Phytopathology. 2003; 93(11): 1452–9. Epub 2008/10/24. doi: 10.1094/PHYTO.2003.93.11.1452 . 18944075 10.1094/PHYTO.2003.93.11.1452

[8] Yang S, Gu T, Pan C, Feng Z, Ding J, Hang Y, et al Genetic variation of NBS-LRR class resistance genes in rice lines. Theor Appl Genet. 2008; 116(2): 165–77. Epub 2007/10/13. doi: 10.1007/s00122-007-0656-4 . 17932646 10.1007/s00122-007-0656-4

[9] Yang S, Zhang X, Yue JX, Tian D, Chen JQ. Recent duplications dominate NBS-encoding gene expansion in two woody species. Mol Genet Genomics. 2008; 280(3): 187–98. Epub 2008/06/20. doi: 10.1007/s00438-008-0355-0 . 18563445 10.1007/s00438-008-0355-0

[10] Hofberger JA, Zhou B, Tang H, Jones JD, Schranz ME. A novel approach for multi-domain and multi-gene family identification provides insights into evolutionary dynamics of disease resistance genes in core eudicot plants. BMC Genomics. 2014; 15: 966 Epub 2014/11/09. doi: 10.1186/1471-2164-15-966 25380807 10.1186/1471-2164-15-966 PMC4289383

[11] Li J, Ding J, Zhang W, Zhang Y, Tang P, Chen JQ, et al Unique evolutionary pattern of numbers of gramineous NBS-LRR genes. Mol Genet Genomics. 2010; 283(5): 427–38. Epub 2010/03/11. doi: 10.1007/s00438-010-0527-6 . 20217430 10.1007/s00438-010-0527-6

[12] Noir S, Combes MC, Anthony F, Lashermes P. Origin, diversity and evolution of NBS-type disease-resistance gene homologues in coffee trees (Coffea L.). Molecular Genetics & Genomics Mgg. 2001; 265(4): 654–62. 11459185 10.1007/s004380100459

[13] Yu J, Tehrim S, Zhang F, Tong C, Huang J, Cheng X, et al Genome-wide comparative analysis of NBS-encoding genes between *Brassica* species and *Arabidopsis thaliana*. BMC Genomics. 2014; 15: 3 Epub 2014/01/05. doi: 10.1186/1471-2164-15-3 24383931 10.1186/1471-2164-15-3 PMC4008172

[14] Bergelson J, Kreitman M, Stahl EA, Tian D. Evolutionary dynamics of plant R-genes. Science. 2001; 292(5525): 2281–5. Epub 2001/06/26. doi: 10.1126/science.1061337 . 11423651 10.1126/science.1061337

[15] Zhou T, Wang Y, Chen J-Q, Araki H, Jing Z, Jiang K, et al Genome-wide identification of NBS genes in japonica rice reveals significant expansion of divergent non-TIR NBS-LRR genes. Molecular Genetics and Genomics. 2004; 271: 402–415. doi: 10.1007/s00438-004-0990-z 15014983 10.1007/s00438-004-0990-z

[16] Ding J, Zhang W, Jing Z, Chen J-Q, Tian D. Unique pattern of R-gene variation within populations in *Arabidopsis*. Molecular Genetics and Genomics. 2007; 277: 619 doi: 10.1007/s00438-007-0213-5 17277944 10.1007/s00438-007-0213-5

[17] Yang S, Feng Z, Zhang X, Jiang K, Jin X, Hang Y, et al Genome-wide investigation on the genetic variations of rice disease resistance genes. Plant Molecular Biology. 2006; 62: 181–193. doi: 10.1007/s11103-006-9012-3 16915523 10.1007/s11103-006-9012-3

[18] Tian, Dacheng. Unbalanced gene copy mediated interlocus sequence exchange at the Rpp8 locus in Arabidopsis.

[19] Shen J, Araki H, Chen L, Chen JQ, Tian D. Unique evolutionary mechanism in R-genes under the presence/absence polymorphism in *Arabidopsis thaliana*. Genetics. 2006; 172: 1243 doi: 10.1534/genetics.105.047290 16452149 10.1534/genetics.105.047290 PMC1456222

[20] Zhu Y, Chen H, Fan J, Wang Y, Li Y, Chen J, et al Genetic diversity and disease control in rice. Nature. 2000; 406: 718–722. doi: 10.1038/35021046 10963595 10.1038/35021046

[21] Gu K, Yang B, Tian D, Wu L, Wang D, Sreekala C, et al R gene expression induced by a type-III effector triggers disease resistance in rice. Nature. 2005; 435: 1122–1125. doi: 10.1038/nature03630 15973413 10.1038/nature03630

[22] Römer P, Hahn S, Jordan T, Strauß T, Bonas U, Lahaye T. Plant pathogen recognition mediated by promoter activation of the pepper *Bs3* resistance gene. Science. 2007; 318: 645 doi: 10.1126/science.1144958 17962564 10.1126/science.1144958

[23] Strauß T, van Poecke RMP, Strauß A, Römer P, Minsavage GV, Singh S, et al RNA-seq pinpoints a *Xanthomonas* TAL-effector activated resistance gene in a large-crop genome. Proceedings of the National Academy of Sciences. 2012; 109: 19480–19485. doi: 10.1073/pnas.1212415109 23132937 10.1073/pnas.1212415109 PMC3511116

[24] Tian D, Yin Z. The rice TAL effector-dependent resistance protein XA10 triggers cell death and calcium depletion in the endoplasmic reticulum. Plant Cell. 2014; 26: 497–515. doi: 10.1105/tpc.113.119255 24488961 10.1105/tpc.113.119255 PMC3963592

[25] Wang C, Zhang X, Fan Y, Gao Y, Zhu Q, Zheng C, et al XA23 is an executor R protein and confers broad-spectrum disease resistance in rice. Molecular plant. 2015; 8(2): 290–302. Epub 2015/01/27. doi: 10.1016/j.molp.2014.10.010 . 25616388 10.1016/j.molp.2014.10.010

[26] Ellur RK, Khanna A, Yadav A, Pathania S, Rajashekara H, Singh VK, et al Improvement of Basmati rice varieties for resistance to blast and bacterial blight diseases using marker assisted backcross breeding. Plant Science. 2016; 242: 330–41. doi: 10.1016/j.plantsci.2015.08.020 26566849 10.1016/j.plantsci.2015.08.020

[27] Gu K, Tian D, Yang F, Wu L, Sreekala C, Wang D, et al High-resolution genetic mapping of *Xa27(t)*, a new bacterial blight resistance gene in rice, *Oryza sativa* L. Theoretical and Applied Genetics. 2004; 108: 800–807. doi: 10.1007/s00122-003-1491-x 15118822 10.1007/s00122-003-1491-x

[28] Wu L, Goh ML, Sreekala C, Yin Z. XA27 depends on an amino-terminal signal-anchor-like sequence to localize to the apoplast for resistance to *Xanthomonas oryzae* pv. *oryzae*. Plant Physiology. 2008; 148: 1497–1509. doi: 10.1104/pp.108.123356 18784285 10.1104/pp.108.123356 PMC2577279

[29] Yoshimura A, Mew TW, Khush GS, Omura T. Inheritance of resistance to bacterial blight in rice cultivar Cas 209. Phytopathology. 1983; 73: 1409–1412.

[30] Hopkins CM, White FF, Choi SH, Guo A, Leach JE. Identification of a family of avirulence genes from *Xanthomonas oryzae* pv. *oryzae*. Mol Plant Microbe Interact. 1992; 5: 451–459. 1335800 10.1094/mpmi-5-451

[31] Zhang Q, Wang CL, Zhao KJ, Zhao YL, Caslana VC, Zhu XD, et al The effectiveness of advanced rice lines with new resistance gene *Xa23* to rice bacterial blight. Rice Genet Newsl. 2001; 18: 71–72.

[32] Wang CL, Qin TF, Yu HM, Zhang XP, Che JY, Gao Y, et al The broad bacterial blight resistance of rice line CBB23 is triggered by a novel transcription activator-like (TAL) effector of *Xanthomonas oryzae* pv. *oryzae*. Mol Plant Pathol. 2014; 15(4): 333–41. Epub 2013/11/30. doi: 10.1111/mpp.12092 . 24286630 10.1111/mpp.12092 PMC6638860

[33] Alexandrov N, Tai S, Wang W, Mansueto L, Palis K, Fuentes RR, et al SNP-Seek database of SNPs derived from 3000 rice genomes. Nucleic Acids Research. 2015; 43(Database issue): 1023–7. 10.1093/nar/gku1039 PMC4383887 25429973

[34] The 3,000 rice genomes project. Gigascience. 2014; 3: 7 Epub 2014/05/30. doi: 10.1186/2047-217X-3-7 24872877 10.1186/2047-217X-3-7 PMC4035669

[35] Doyle J. Isolation of Plant DNA from fresh tissue. Focus. 1990; 12: 13–5.

[36] Larkin MA, Blackshields G, Brown NP, Chenna R, McGettigan PA, McWilliam H, et al Clustal W and Clustal X version 2.0. Bioinformatics. 2007; 23(21): 2947–8. Epub 2007/09/12. doi: 10.1093/bioinformatics/btm404 . 17846036 10.1093/bioinformatics/btm404

[37] Alzohairy AM. BioEdit: An important software for molecular biology. Gerf Bulletin of Biosciences. 2011; 2(1): 60–1.

[38] Hall TA, editor BioEdit: a user-friendly biological sequence alignment editor and analysis program for Windows 95/98/NT. Nucl Acids Symp Ser; 1999.

[39] Librado P, Rozas J. DnaSP v5: a software for comprehensive analysis of DNA polymorphism data. Bioinformatics. 2009; 25(11): 1451–2. doi: 10.1093/bioinformatics/btp187 19346325 10.1093/bioinformatics/btp187

[40] Nei M, Li WH. Mathematical model for studying genetic variation in terms of restriction endonucleases. Proceedings of the National Academy of Science. 1979; 76(10): 5269–73. 10.1073/pnas.76.10.5269 PMC413122 291943

[41] Watterson GA. On the number of segregating sites in genetical models without recombination. Theoretical population biology. 1975; 7(2): 256–76. 1145509 10.1016/0040-5809(75)90020-9

[42] Tajima F. Statistical method for testing the neutral mutation hypothesis by DNA polymorphism. Genetics. 1989; 123(3): 585–95. 2513255 10.1093/genetics/123.3.585 PMC1203831

[43] Fu YX, Li WH. Statistical tests of neutrality of mutations. Genetics. 1993; 133(3): 693–709. 8454210 10.1093/genetics/133.3.693 PMC1205353

[44] Bandelt HJ, Forster P, Rohl A. Median-joining networks for inferring intraspecific phylogenies. Molecular biology and evolution. 1999; 16(1): 37–48. 10331250 10.1093/oxfordjournals.molbev.a026036

[45] Kauffman HE, Reddy APK, Hsieh SPY, Merca SD. An improved technique for evaluating resistance of rice varieties to *Xanthomonas oryzae* pv. *oryzae*. Plant Disease Reporter. 1973; 57(6): 537–41.

[46] Zhang XP, Wang CL, Zheng CK, et al HrcQ is necessary for *Xanthomonas oryzae* pv. *oryzae* HR-induction in non-host tobacco and pathogenicity in host rice. Crop Journal. 2013; 1(2): 143–50.

[47] May RM, Anderson RM. Parasite-host coevolution In: Futuyama DJ, Slatkin M (eds) Coevolution. Sinauer Associates, Sunderland, 1983; pp. 186–206.

[48] Rose LE, Michelmore RW, Langley CH. Natural variation in the Pto disease resistance gene within species of wild tomato (Lycopersicon). II. Population genetics of *Pto*. Genetics. 2007; 175: 1307–1319. doi: 10.1534/genetics.106.063602 17179076 10.1534/genetics.106.063602 PMC1840093

[49] Zhu Q, Zheng X, Luo J, Gaut BS, Ge S. Multilocus analysis of nucleotide variation of Oryza sativa and its wild relatives: severe bottleneck during domestication of rice. Molecular Biology and Evolution. 2007; 24: 875–888. doi: 10.1093/molbev/msm005 17218640 10.1093/molbev/msm005

[50] Eyre Walker A, Gaut RL, Hilton H, Feldman DL, Gaut BS. Investigation of the bottleneck leading to the domestication of maize. Proc Natl Acad Sci USA. 1998; 95: 4441–4446. . 9539756 10.1073/pnas.95.8.4441 PMC22508

[51] Buckler ES, Thornsberry JM, Kresovich S. Molecular diversity, structure and domestication of grasses. Genet Res. 2001; 77: 213–218. . 11486504 10.1017/s0016672301005158

[52] Small RL, Wendel JF. Copy number lability and evolutionary dynamics of the *Adh* gene family in diploid and tetraploid cotton (Gossypium). Genet 2000; 155: 1913–1926. . 10924485 10.1093/genetics/155.4.1913 PMC1461218

[53] Rose LE, Bittner Eddy PD, Langley CH, Holub EB, Michelmore RW, Beynon JL. The maintenance of extreme amino acid diversity at the disease resistance gene, *RPP13*, in *Arabidopsis thaliana*. Genet. 2004; 166: 1517–1527. . 15082565 10.1534/genetics.166.3.1517 PMC1470773

[54] Hummel AW, Doyle EL, Bogdanove AJ. Addition of transcription activator-like effector binding sites to a pathogen strain-specific rice bacterial blight resistance gene makes it effective against additional strains and against bacterial leaf streak. New Phytol. 2012; 195(4): 883–93. Epub 2012/07/04. doi: 10.1111/j.1469-8137.2012.04216.x . 22747776 10.1111/j.1469-8137.2012.04216.x

[55] Romer P, Recht S, Lahaye T. A single plant resistance gene promoter engineered to recognize multiple TAL effectors from disparate pathogens. Proc Natl Acad Sci U S A. 2009; 106(48): 20526–31. Epub 2009/11/17. doi: 10.1073/pnas.0908812106 19910532 10.1073/pnas.0908812106 PMC2776607

[56] Zhou Y-L, Xu J-L, Zhou S-C, Yu J, Xie X-W, Xu M-R, et al Pyramiding Xa23 and Rxo1 for resistance to two bacterial diseases into an elite *indica* rice variety using molecular approaches. Molecular breeding: new strategies in plant improvement. 2009; 23: 279–287. doi: 10.1007/s11032-008-9232-0

[57] Huang B, Xu J, Hou M, Ali J, Mou T. Introgression of bacterial blight resistance genes Xa7, Xa21, Xa22 and Xa23 into hybrid rice restorer lines by molecular marker-assisted selection. Euphytica: Netherlands journal of plant breeding. 2012; 187: 449–459. doi: 10.1007/s10681-012-0758-1

[58] Kover PX, Schaal BA. Genetic variation for disease resistance and tolerance among *Arabidopsis thaliana* accessions. Proc Natl Acad Sci U S A. 2002; 99(17): 11270–4. Epub 2002/08/13. doi: 10.1073/pnas.102288999 12172004 10.1073/pnas.102288999 PMC123246

