# Supplementary material for: Promoter variants of Xa23 alleles affect bacterial blight resistance and evolutionary pattern
Source: PLoS One. 2017 Oct 5;12(10):e0185925. doi: 10.1371/journal.pone.0185925 (PMC5628896; doi:10.1371/journal.pone.0185925)
Supplement: S1 Fig — The 1720-bp sequence of Xa23 allele in CBB23 was used as the reference. The numbers at right side indicate nucleotide positions of the CBB23 and JG30 sequences. The 28-bp EBEAvrXa23 and 34-bp ebeJG30 are highlighted in green. The 7-bp polymorphic nucleotides (6-bp insertion and 1-bp substitution) in JG30 are highlighted in purple. The coding regions of Xa23/xa23 alleles in CBB23 and JG30 are both highlighted in blue. The primers are used for amplifying the alignment regions covering entire EBE/ebe and condign regions. The arrows indicate the primers and their amplification directions. (PDF) [file pone.0185925.s001.pdf]

```
             F1
JG30         CTGAGGTAGCTGCCACGTCAGCTAGGGGATCGGCCCGGCCGCCACGGTGG     50
CBB23        --------------------------------------------------     50

JG30         CACAATGTCAGCGCCAGTCCCGTTGGCGCTGACACGGCCAACGTCAGCGC    100
CBB23        --------------------------------------------------    100

JG30         CAATGTGTTTGGCGCTGAGGCGACGGCCTATTTTTGGTTGAAGTTTTTGG    150
CBB23        --------------------------------------------------    150

JG30         CAGGGGTTAGTTTCGAAATAAGTTTTCCAAAAGGGTCAATTTGTCAAAAA    200
CBB23        --------------------------------------------------    200
                   F2
JG30         AACGGCTCGTCCCTGAGTCAAAGTCTTCCCTATTAAATTATGCGGCATCA    250
CBB23        --------------------------------------------------    250

JG30         CTAACATCAGCTACTATAAAAGTCCCTTCCGCGTCACTAACATCAGCTAC    300
CBB23        --------------------------------------------------    300
                                        ebe_JG30
JG30         TATAAAAGTCCCTTCCGCATCACTAACATCTTCCTCCCGCATCACTAACA    350
CBB23        -------------TCCG......aAACATCTTCCTCCCGCATCACTA---    344
              F3                    EBE_AvrXa23
JG30         TCAGCTTCTATAAAAGCCCTTCCTTGTTGCATCATCTCAAGGAGCTGCAA    400
CBB23        --------------------------------------------------    394
                                  R5                   F4
JG30         GCACTTCCTCTCTGGCAGCACTTCCTCATCTCAAGGAGTTGCAAATGTTG    450
CBB23        --------------------------------------------------    444

JG30         CATCATCTCAAGGAGCTGGCAGCCGTAGCCGGTATACACATGATCCTCAT    500
CBB23        --------------------------------------------------    494
                                  R4
JG30         CTACCTCTGCCGCTTTCTCCTCCGCCGCAGCCGCAACGTATTATTCACCG    550
CBB23        --------------------------------------------------    544

JG30         TTTCCAACAGCCTCCGTTTTCGCCTCAAGGTATTAACTGTATTGTTGTAC    600
CBB23        --------------------------------------------------    594

JG30         ATATGTCTCTCGGTCATGCTGTTCTACCTGTTTGGCTCCATCATGCCGCT    650
CBB23        --------------------------------------------------    644

JG30         GCCGCCGTGGGGCCTCGTGGTCGGTTGGGTCATGGCCCTCATCGCCGTCG    700
CBB23        --------------------------------------------------    694
                                  R3
JG30         AGCTCGCCTACGCCTTCATCTTTCCATATAGCTTTCGCTACATCGCTGAC    750
CBB23        --------------------------------------------------    744
                                             R2
JG30         AACGACGACGACAAGATGGTTATTCTCCCTGTTTAAGCCTTCAGGGCCTA    800
CBB23        --------------------------------------------------    794

JG30         TATATATAGTATATATATAAAGCCTTCCATACTGTCTCTTCAATAAAGGC    850
CBB23        --------------------------------------------------    844

JG30         TAGCTTGTGTTGTGAGTTGTATCTGTGTACGTATTTTGTTTGGTTGGTTA    900
CBB23        --------------------------------------------------    894

JG30         TATATTGTCACGTAGGTATGCCATATATATATGTATTGCTGTATTTATAT    950
CBB23        --------------------------------------------------    944

JG30         TTGTTACTATCTTTTGTTTTTCAGATAATAAAATTCAGCCAGCTTTGCTT   1000
CBB23        --------------------------------------------------    994

JG30         GCTTCGTCGTACGTGTATGCTCATCATATCCTCATCCATCAGCTGCTCAT   1050
CBB23        --------------------------------------------------   1044

JG30         AGCTAGCTGGGCCGTGTTATATGTGTGTAGTGATCAGTCACATCCATGTA   1100
CBB23        --------------------------------------------------   1094
                                 R1
JG30         TTTCATCCCATGTATGTTAGTTTGTTTTTCATTTTTGAAAGAAAACATGC   1150
CBB23        --------------------------------------------------   1144

JG30         ATATATGTTATAACTCAAAGTTTTGAGATGAATTTGATCATCTAATTTAT   1200
CBB23        --------------------------------------------------   1194

JG30         TTCCGTTTTGAATTATTCTGCTCTGTTGTTGCAAACTTTTTTTTTTATGT   1250
CBB23        --------------------------------------------------   1244

JG30         TGGGACACTTTCTCCTCGGTTTGATTGTTATATTGAATTCGTCGATTCCA   1300
CBB23        --------------------------------------------------   1294

JG30         GCTAGTATATATTTGCTAGTTTCACTCAGACAATCATGCCTATTTGCTGA   1350
CBB23        --------------------------------------------------   1344

JG30         TCAGGAACAATTGAAAACAACCATTACAGAGCCAGACGAAATTAATTAAT   1400
CBB23        --------------------------------------------------   1394

JG30         TTACACTCACCAATTATTCATCATCACATCTCTAAACATCGAATTCTGGG   1450
CBB23        --------------------------------------------------   1444

JG30         ATTCCATCGTTCGTCCCCTTCCCGCAGCCGTCGGATCGCGGATCGACGGT   1500
CBB23        --------------------------------------------------   1494

JG30         GGCAGATCGCTTCGTTGAACGTTTTTGTAATATACCATTTCTTGAGGGGG   1550
CBB23        --------------------------------------------------   1544

JG30         GTTTATGCAAAATATCTCTATCCCTTACCTCCTCCTACCTCGCCGGCGTG   1600
CBB23        --------------------------------------------------   1594

JG30         ACGCTCTCCTCCCTCACTCCCATCCGCCGCCGGCGAGGCCCTCTTCGCCG   1650
CBB23        --------------------------------------------------   1644

JG30         CCGTGTCCTCCTCGTCGTCGCTGTATGGGGCGAGGCCGCGGCCCGCGAGG   1700
CBB23        --------------------------------------------------   1694

JG30         GGCTCGGTGGCGGCGCGGGGGGATGG                        1726
CBB23        --------------------------                        1720
```

S1 Fig. Alignment of nucleotide sequences between the resistant *Xa23* allele in CBB23 and susceptible *xa23* allele in JG30. The 1720-bp sequence of *Xa23* allele in CBB23 was used as the reference. The numbers at right side indicate nucleotide positions of the CBB23 and JG30 sequences. The 28-bp EBE_{AvrXa23} and 34-bp ebe_{JG30} are highlighted in green. The 7-bp polymorphic nucleotides (6-bp insertion and 1-bp substitution) in JG30 are highlighted in purple. The coding regions of *Xa23/xa23* alleles in CBB23 and JG30 are both highlighted in blue. The primers are used for amplifying the alignment regions covering entire *EBE/ebe* and condign regions. The arrows indicate the primers and their amplification directions.
