# Supplementary material for: Promoter variants of Xa23 alleles affect bacterial blight resistance and evolutionary pattern
Source: PLoS One. 2017 Oct 5;12(10):e0185925. doi: 10.1371/journal.pone.0185925 (PMC5628896; doi:10.1371/journal.pone.0185925)
Supplement: S2 Fig — The multiple sequence alignment was constructed using CLC Sequence Viewer 7 program. The Xa23 in CBB23 is used as a reference. The different residues are shown in red. The consensus sequence of all the Xa23/xa23 alleles along with percentage conservation of residue is also shown. The numbers at right side indicate the length of each sequence. (PDF) [file pone.0185925.s002.pdf]

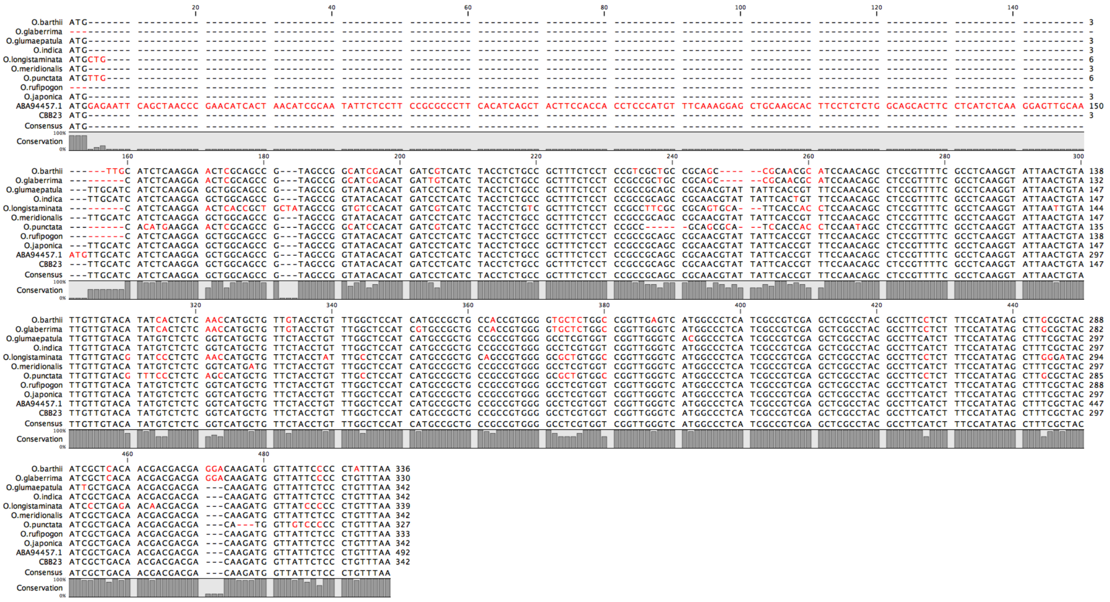

S2 Fig. Comparison of coding regions of *Xa23/xa23* alleles in *Oryza* species. The multiple sequence alignment was constructed using CLC Sequence Viewer 7 program. The *Xa23* in CBB23 is used as a reference. The different residues are shown in red. The consensus sequence of all the *Xa23/xa23* alleles along with percentage conservation of residue is also shown. The numbers at right side indicate the length of each sequence.
