# Supplementary material for: Promoter variants of Xa23 alleles affect bacterial blight resistance and evolutionary pattern
Source: PLoS One. 2017 Oct 5;12(10):e0185925. doi: 10.1371/journal.pone.0185925 (PMC5628896; doi:10.1371/journal.pone.0185925)
Supplement: S3 Fig — These 18 representative rice accessions (8 indica, 3 japonica and 7 wild relatives) were inoculated with X. oryzae pv. oryzae strain PXO99A using leaf-clipping method and bacterial blight lesions were measured 14 days after artificial inoculation. Y-axis is showing the lesion length. The Pro-A, B, C, D and E at the top indicate the five haplotypes of Xa23/xa23 alleles. W, wild rice; I, indica; J, japonica; R, resistant; S, susceptible. (PDF) [file pone.0185925.s003.pdf]

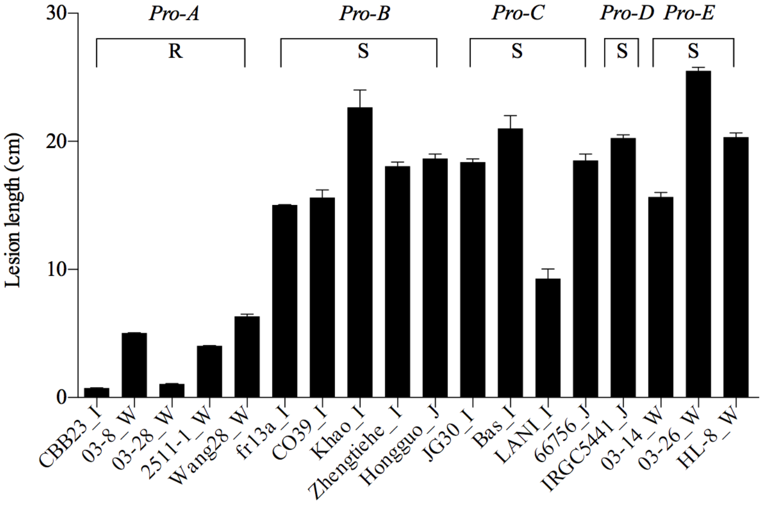

S3 Fig. Disease responses of five haplotypes of *Xa23/xa23* alleles to PXO99^{A}.

These 18 representative rice accessions (8 *indica,* 3 *japonica* and 7 wild relatives) were inoculated with *X. oryzae* pv. *oryzae* strain PXO99^{A} using leaf-clipping method and bacterial blight lesions were measured 14 days after artificial inoculation. Y-axis is showing the lesion length. The *Pro-A*, *B*, *C*, *D* and *E* at the top indicate the five haplotypes of *Xa23/xa23* alleles.

W, wild rice; I, *indica*; J, *japonica*; R, resistant; S, susceptible.
