# Supplementary material for: Promoter variants of Xa23 alleles affect bacterial blight resistance and evolutionary pattern
Source: PLoS One. 2017 Oct 5;12(10):e0185925. doi: 10.1371/journal.pone.0185925 (PMC5628896; doi:10.1371/journal.pone.0185925)
Supplement: S1 Table — (PDF) [file pone.0185925.s004.pdf]

S1 Table. Summary of rice used for polymorphism and haplotype analysis

No.	Variety/Accession	Subspecies/Species	Origin	Haplotype of entire gene	Haplotype of promoter
1	CBB23	*indica*	China	H2	*Pro-A*
2	JG30	*indica*	China	H5	*Pro-C*
3	93-11	*indica*	China	H3	*Pro-B*
4	CO 39	*indica*	India	H3	*Pro-B*
5	fr 13 a	*indica*	India	H3	*Pro-B*
6	da 16	*indica*	Bangladesh	H3	*Pro-B*
7	t 1	*indica*	India	H3	*Pro-B*
8	jhona 349	*indica*	India	H3	*Pro-B*
9	LANI KHAMA	*indica*	Bangladesh	H5	*Pro-C*
10	Basmati 1	*indica*	Pakistan	H5	*Pro-C*
11	MADHABSAR	*indica*	Bangladesh	H3	*Pro-B*
12	Phudugey	*indica*	Bhutan	H8	*Pro-E*
13	Zhengtiehe	*indica*	Fujian, China	H3	*Pro-B*
14	Khao Dawk Mali 105	*indica*	Thailand	H3	*Pro-B*
15	Kokko	*indica*	Taiwan	H5	*Pro-C*
16	Mayang Tapang	*indica*	Malaysia	H3	*Pro-B*
17	Natpyihmwe	*indica*	Myanmar	H3	*Pro-B*
18	45849line	*indica*	India	H3	*Pro-B*
19	CO18	*indica*	India	H3	*Pro-B*
20	5024S	*indica*	China	H5	*Pro-C*
21	SHADA BORO	*japonica*	Bangladesh	H3	*Pro-B*
22	N 22	*japonica*	India	H3	*Pro-B*
23	Nipponbare	*japonica*	Japan	H8	*Pro-E*
24	Hongguo	*japonica*	Heilongjiang, China	H3	*Pro-B*
25	66756	*japonica*	USA	H5	*Pro-C*
26	n 22	*japonica*	India	H4	*Pro-C*
27	jc 1	*japonica*	India	H5	*Pro-C*
28	IRGC 5441	*japonica*	Taiwan	H7	*Pro-D*
29	Cuba 65	*japonica*	Cuba	H5	*Pro-C*

Continued

30	81984	*O. rufipogon*	Guangxi, China	H5	*Pro-C*
31	106452	*O. rufipogon*	Guangxi, China	H3	*Pro-B*
32	HL-2	*O. rufipogon*	Guangdong, China	H8	*Pro-E*
33	HL-8	*O. rufipogon*	Guangdong, China	H8	*Pro-E*
34	HL-39	*O. rufipogon*	Guangdong, China	H8	*Pro-E*
35	HL-48	*O. rufipogon*	Guangdong, China	H8	*Pro-E*
36	DX-22	*O. rufipogon*	Hainan, China	H8	*Pro-E*
37	DX-28	*O. rufipogon*	Hainan, China	H8	*Pro-E*
38	DX-54	*O. rufipogon*	Hainan, China	H8	*Pro-E*
39	GZ-7	*O. rufipogon*	Guangdong, China	H8	*Pro-E*
40	GZ-104	*O. rufipogon*	Guangdong, China	H8	*Pro-E*
41	GZ-117	*O. rufipogon*	Guangdong, China	H9	*Pro-E*
42	SX-12	*O. rufipogon*	Guangdong, China	H8	*Pro-E*
43	SX-16	*O. rufipogon*	Guangdong, China	H9	*Pro-E*
44	FG-12	*O. rufipogon*	Guangdong, China	H9	*Pro-E*
45	FG-19	*O. rufipogon*	Guangdong, China	H8	*Pro-E*
46	03-8	*O. rufipogon*	Guangxi, China	H1	*Pro-A*
47	03-9	*O. rufipogon*	Guangxi, China	H1	*Pro-A*
48	03-66	*O. rufipogon*	Guangxi, China	H1	*Pro-A*
49	Wang13	*O. rufipogon*	Guangxi, China	H1	*Pro-A*
50	2511-2	*O. rufipogon*	Guangxi, China	H6	*Pro-C*
51	2511-1	*O. rufipogon*	Guangxi, China	H1	*Pro-A*
52	Wang28	*O. rufipogon*	Guangxi, China	H1	*Pro-A*
53	03-14	*O. rufipogon*	Guangxi, China	H8	*Pro-E*
54	03-15	*O. rufipogon*	Guangxi, China	H1	*Pro-A*
55	03-16	*O. rufipogon*	Guangxi, China	H8	*Pro-E*
56	03-19	*O. rufipogon*	Guangxi, China	H1	*Pro-A*
57	03-20	*O. rufipogon*	Guangxi, China	H1	*Pro-A*
58	03-26	*O. rufipogon*	Guangxi, China	H8	*Pro-E*
59	03-27	*O. rufipogon*	Guangxi, China	H1	*Pro-A*

Continued

60	03-28	*O. rufipogon*	Guangxi, China	H1	*Pro-A*
61	03-104	*O. rufipogon*	Guangxi, China	H1	*Pro-A*
62	04-72	*O. rufipogon*	Guangxi, China	H1	*Pro-A*
63	04-102	*O. rufipogon*	Guangxi, China	H1	*Pro-A*
64	04-103	*O. rufipogon*	Guangxi, China	H8	*Pro-E*
65	04-108S	*O. rufipogon*	Guangxi, China	H8	*Pro-E*
66	04-110	*O. rufipogon*	Guangxi, China	H1	*Pro-A*
67	KHM08-37	*O. rufipogon*	Cambodia	H3	*Pro-B*
68	KHM08-51	*O. rufipogon*	Cambodia	H3	*Pro-B*
69	NEP09-79	*O. rufipogon*	Nepal	H3	*Pro-B*
70	BC2701	*O. rufipogon*	Hainan, China	H3	*Pro-B*
71	103416	*O. nivara*	unknown	H4	*Pro-C*
72	105320	*O. nivara*	unknown	H3	*Pro-B*
73	105789	*O. nivara*	unknown	H3	*Pro-B*
74	81801	*O. nivara*	Indonesia	H4	*Pro-C*
75	81865	*O. nivara*	India	H3	*Pro-B*
76	81881	*O. nivara*	India	H3	*Pro-B*
77	VN04-9	*O. nivara*	Laos	H3	*Pro-B*
78	VN04-10	*O. nivara*	Laos	H3	*Pro-B*
79	VN07-10	*O. nivara*	Laos	H3	*Pro-B*
80	VN07-00	*O. nivara*	Laos	H3	*Pro-B*
81	DP-2	*O. officinalis wall*	Guangxi, China	H6	*Pro-C*
82	DP-7	*O. officinalis wall*	Guangxi, China	H6	*Pro-C*
83	DP-14	*O. officinalis wall*	Guangxi, China	H6	*Pro-C*
84	DP-15	*O. officinalis wall*	Guangxi, China	H6	*Pro-C*
85	DP-16	*O. officinalis wall*	Guangxi, China	H6	*Pro-C*
86	DP-25	*O. officinalis wall*	Guangxi, China	H6	*Pro-C*
87	DP-31	*O. officinalis wall*	Guangxi, China	H6	*Pro-C*
88	DP-33	*O. officinalis wall*	Guangxi, China	H10	*Pro-E*
89	DP-35	*O. officinalis wall*	Guangxi, China	H6	*Pro-C*

Continued

90	DP-38	*O. officinalis wall*	Guangxi, China	H6	*Pro-C*
91	DP-42	*O. officinalis wall*	Guangxi, China	H6	*Pro-C*
92	DP-46	*O. officinalis wall*	Guangxi, China	H6	*Pro-C*
93	DP-48	*O. officinalis wall*	Guangxi, China	H10	*Pro-E*
94	DP-50	*O. officinalis wall*	Guangxi, China	H6	*Pro-C*
95	94	*O. alta swallen*	Guangxi, China	H5	*Pro-C*
96	84	*O. glumaepatula*	Guangxi, China	H5	*Pro-C*
97	03-101	*O. latifolia desy*	Guangxi, China	H6	*Pro-C*

"unknown" indicates no detailed geographic information.
