# Supplementary material for: Promoter variants of Xa23 alleles affect bacterial blight resistance and evolutionary pattern
Source: PLoS One. 2017 Oct 5;12(10):e0185925. doi: 10.1371/journal.pone.0185925 (PMC5628896; doi:10.1371/journal.pone.0185925)
Supplement: S2 Table — (PDF) [file pone.0185925.s005.pdf]

S2 Table. Primers used for amplification and sequencing in this research

Primer name	Forward primer sequence (5'-3')	Description
F1	CTGAGGTAGCTGCCACGTCAGCTAGGG	amplification/sequencing
F2	TCGTCCCTGAGTCAAAGTCTTCCCT	amplification/sequencing
F3	TCCGAAACATCTTCCTCCCGCATCACTA	amplification/sequencing
F4	ATGTTGCATCATCTCAAGGAGCT	amplification/sequencing
R1	CATCCCATGTATGTTAGTTTGT	amplification/sequencing
R2	TTAAACAGGGAGAATAACCATCTTG	amplification/sequencing
R3	TGGAAAGATGAAGGCGTAGGCGAGC	amplification/sequencing
R4	GGCGGAGGAGAAAGCGGCAGAGGTA	amplification/sequencing
R5	ATGAGGAAGTGCTGCCAGAGAGGAA	amplification/sequencing
Xa23-9311-F	GTGGGGTGTGAGGGGTAGGAGAGGG	amplification/sequencing
Xa23-9311-R1	AGGAAGTGCTGCCAGAGAGGAAGTG	amplification/sequencing
Xa23-9311-R2	GCGGCGGAGGAGAAAGCGGCAGAGG	amplification/sequencing
