# Supplementary material for: Promoter variants of Xa23 alleles affect bacterial blight resistance and evolutionary pattern
Source: PLoS One. 2017 Oct 5;12(10):e0185925. doi: 10.1371/journal.pone.0185925 (PMC5628896; doi:10.1371/journal.pone.0185925)
Supplement: S3 Table — (PDF) [file pone.0185925.s006.pdf]

S3 Table. The SNPs in coding regions of *Xa23* alleles and corresponding bacterial blight resistance phenotypes

Variety	Species	Identity (%)	S1 (24)	S2 (105)	Haplotype	Genotype	Phenotype
CBB23	*indica*	100	G	C	H2	A1	R
JG30	*indica*	100	G	C	H5	A1	S
LANI KHAMA	*indica*	100	G	C	H5	A1	S
5024S	*indica*	100	G	C	H5	A1	S
Zhengtiehe	*indica*	99	G	T	H3	A2	S
Khao Dawk Mali 105	*indica*	99	G	T	H3	A2	S
fr 13 a	*indica*	99	G	T	H3	A2	S
CO 39	*indica*	99	G	T	H3	A2	S
Nipponbare	*japonica*	100	G	C	H8	A1	S
66756	*japonica*	100	G	C	H5	A1	S
Hongguo	*japonica*	99	G	T	H3	A2	S
03-14	*O. rufipogon*	100	G	C	H8	A1	S
03-15	*O. rufipogon*	99	A	C	H1	A3	R
03-16	*O. rufipogon*	100	G	C	H8	A1	S
03-26	*O. rufipogon*	100	G	C	H8	A1	S
03-27	*O. rufipogon*	99	A	C	H1	A3	R
03-66	*O. rufipogon*	99	A	C	H1	A3	R
04-108S	*O. rufipogon*	100	G	C	H8	A1	S
Wang13	*O. rufipogon*	99	A	C	H1	A3	R
Wang28	*O. rufipogon*	99	A	C	H1	A3	R
2511-2	*O. rufipogon*	99	A	C	H6	A3	S
03-101	*O. latifolia desy*	99	A	C	H6	A3	S

R, resistant; S, susceptible.
